# Effect of Educational Brochures on Preprocedural Anxiety in Pediatric Endoscopy: A Randomized Controlled Trial

**DOI:** 10.5152/tjg.2025.25144

**Published:** 2025-09-10

**Authors:** Selin Erel, Aslıhan Güleç Kılıç, Nuray Camgöz Eryılmaz, Ülgen Öztürk Toyran, Ödül Eğritaş Gürkan

**Affiliations:** 1 Department of Anesthesiology and Reanimation, Gazi University Faculty of Medicine, Ankara, Türkiye; 2 Clinic of Anesthesiology and Reanimation, Prof. Dr. Cemil Taşçıoğlu City Hospital, İstanbul, Türkiye; 3 Department of Pediatric Gastroenterology, Gazi University Faculty of Medicine, Ankara, Türkiye

**Keywords:** Anesthesia, anxiety, deep sedation, endoscopy, pediatrics

## Abstract

**Background/Aims:**

Preoperative anxiety is a common and significant issue in pediatric patients undergoing endoscopic interventions. The evaluation aimed to determine whether age-appropriate educational brochures could reduce anxiety in pediatric patients and their caregivers undergoing outpatient endoscopic procedures with sedation.

**Materials and Methods:**

Pediatric patients and their caregivers were randomly assigned to either a control group (standard verbal information only) or an intervention group (standard verbal information plus an age-appropriate educational brochure). On the day of the procedure, pediatric anxiety was assessed using the Modified Yale Preoperative Anxiety Scale (m-YPAS), whereas caregiver anxiety was measured using the Amsterdam Preoperative Anxiety and Information Scale (APAIS).

**Results:**

A total of 252 pediatric patients (age, 3-17 years; American Society of Anesthesiologists score, I-II) and their caregivers were recruited. Of these, 174 formed the control group and 78 received an educational brochure intervention. The demographic characteristics were similar across the groups, except for caregiver age. While there were no significant between-group differences in caregiver APAIS scores, pediatric patients in the brochure group exhibited significantly lower m-YPAS scores (*P* < .05) than those in the control group.

**Conclusion:**

Providing children with age-appropriate educational brochures prior to sedation for endoscopic procedures significantly reduced their preoperative anxiety. This low-cost, easily implemented intervention may help improve procedural experience in pediatric patients and potentially enhance overall clinical outcomes.

**Main Points**

- Preprocedural anxiety is commonly observed in pediatric patients undergoing endoscopic procedures and may impact procedural outcomes.
- Providing educational brochures significantly reduces pediatric preprocedural anxiety but does not have a measurable effect on caregiver anxiety.
- The most frequently reported concern of caregivers related to anesthesia is the fear that their child might not awaken after the procedure.

## Introduction

Anxiety is a widespread and critical concern among pediatric patients undergoing surgical or diagnostic procedures, with studies indicating that up to 70% of these children experience pronounced preoperative distress.^1^ This heightened anxiety often manifests fear, uncooperativeness, and agitation—particularly when children are separated from their parents or confronted with unfamiliar medical settings and equipment. In addition to triggering physiological responses such as elevated heart rate and blood pressure, preoperative anxiety has been linked to increased pain sensitivity, poor compliance, and, in some cases, the cancellation of procedures.^2^ Furthermore, children who exhibit high levels of anxiety before a procedure are at greater risk of postoperative agitation, delirium, and behavioral disturbances—including nightmares, separation anxiety, sleep disorders, and hostility toward healthcare personnel.^3^ These repercussions can persist for months and may adversely influence children’s future interactions with medical services.^4^

Over the past decade, the demand for pediatric procedural sedation outside the operating room has grown, partly due to advancements in radiology, pediatrics, and anesthesiology.^5^ Endoscopy has emerged as a critical tool for diagnosing and treating pediatric gastrointestinal conditions. While sedation-free approaches have been explored for certain procedures, deep sedation or general anesthesia remains the clinical gold standard—particularly for younger patients—to minimize psychological trauma and ensure optimal procedural conditions.^6^However, children with marked anxiety may require higher doses of sedatives, increasing the risk of adverse events such as sedation failure and complications.^7^

To address these challenges, both pharmacological and non-pharmacological strategies have been employed to reduce preoperative anxiety in pediatric populations. These interventions include distraction techniques, such as playing app-based games and engaging in virtual reality experiences, as well as preoperative education programs incorporating educational leaflets and videos.^8^^-^^11^ Moreover, preprocedural anxiety of the caregivers is also a known accompanying factor of pediatric anxiety and similar to pediatric population, education of the parents about upcoming procedure either by reading materials or audiovisual tools may help decrease the parental anxiety.^12^^-^^14^ There are promising findings regarding the use of informational brochures in reducing preoperative anxiety in pediatric patients.^10^^,^^15^^,^^16^However, their potential role in procedures performed outside the operating room and/or under sedation, such as endoscopy, has not yet been investigated in the Turkish population.

This randomized controlled study aimed to assess the efficacy of informational brochures in improving preoperative knowledge and reducing anxiety in children and their parents undergoing outpatient pediatric endoscopy under sedation.

## Materials and Methods

### Study Design

This single-blinded, randomized clinical study was conducted at Gazi University Hospital between January 2022 and February 2023 in children undergoing elective outpatient gastrointestinal endoscopy under sedation, along with their caregivers.

Indications of endoscopy were difficulty swallowing, chronic vomiting, non urgent upper gastrointestinal bleeding lead to melena, blood positivity in stool and/or chronic anemia, chronic abdominal pain, chronic diarrhea, impaired digestion, chronic pallor, or unexplained weight loss. Upper gastrointestinal endoscopy was performed with an Olympus GIF P230 videogastroscope (Olympus Optical Corporation, Tokyo, Japan).

**Figure 1.**
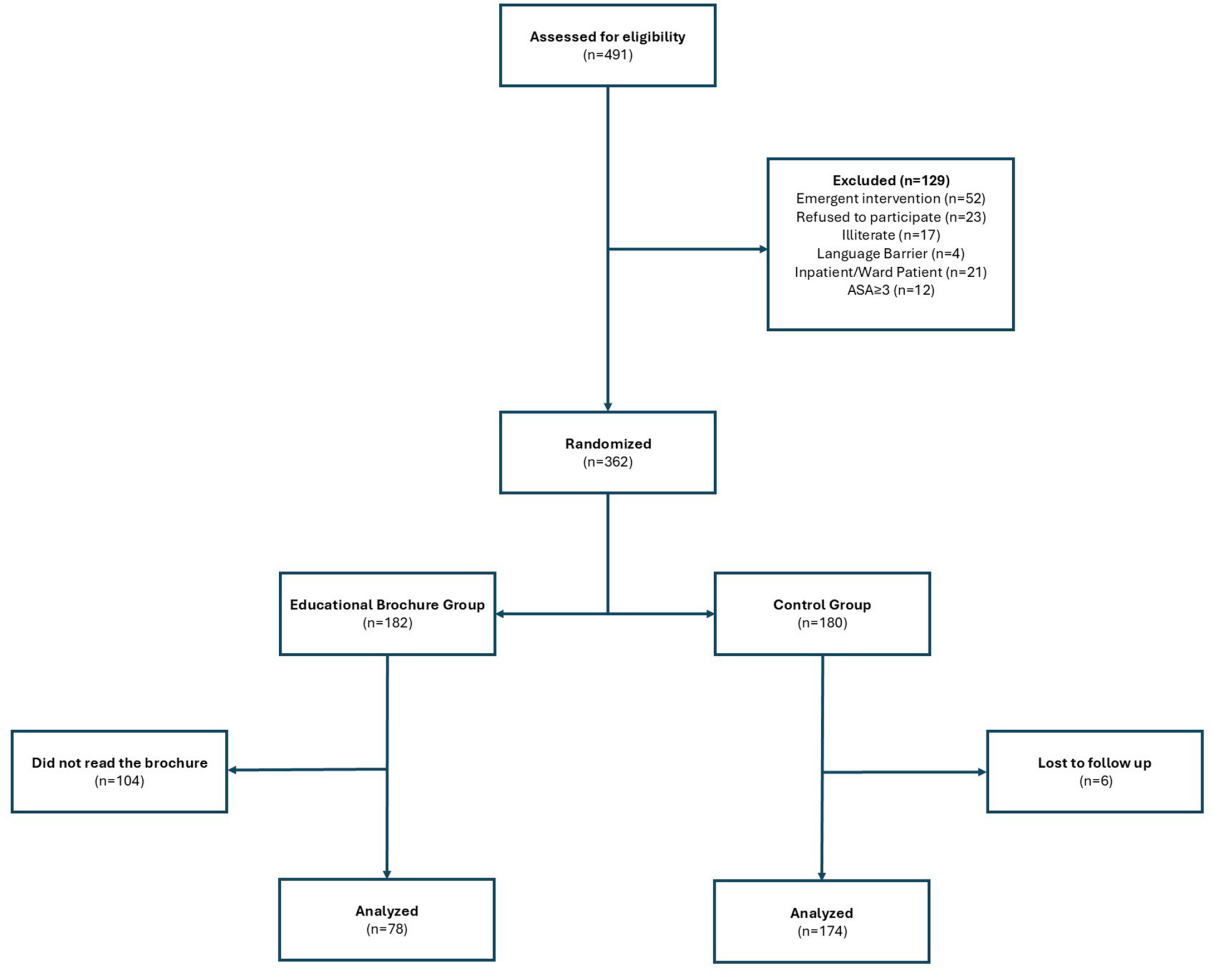
Patient selection and randomization flow chart.

Children aged 3-17 years with an American Society of Anesthesiologists score of I–II, scheduled for an elective endoscopy procedure, and with no prior diagnosis of a neuropsychiatric disease were included in the study, provided they had a Turkish-speaking, literate caregiver. The exclusion criteria included refusal to participate, the presence of an illiterate caregiver, the requiring emergent intervention, urgent endoscopy required due to food impaction or bleeding, current hospitalization in a ward or intensive care unit, and the simultaneous participation of siblings undergoing endoscopy (Figure 1).

### Preanesthetic Visit and Randomization

All pediatric patients scheduled for elective gastrointestinal endoscopy were evaluated by an independent anesthesiologist who was not involved in the study at the preoperative anesthesia assessment outpatient clinic at least 5 days before the planned procedure. Routine preprocedural anesthesia assessments were performed for all patients. Parents and children were informed about the study, and those who provided written informed consent were randomly assigned to either the control or intervention group using a computer-generated randomization process.

- Control group: Patients and their caregivers received standard verbal information about the peri-sedation phase, as routinely provided in the outpatient clinic, without receiving an educational brochure.
- Intervention group*:* Patients received an age-appropriate educational brochure, while their caregivers were provided with the corresponding version for adults. If the child was old enough to read, they were encouraged to read the brochure at home; otherwise, the caregivers were asked to read it. Each parent and child were instructed to read their respective brochures for at least 2 days before the procedure.

### Educational Intervention

**Figure 2.**
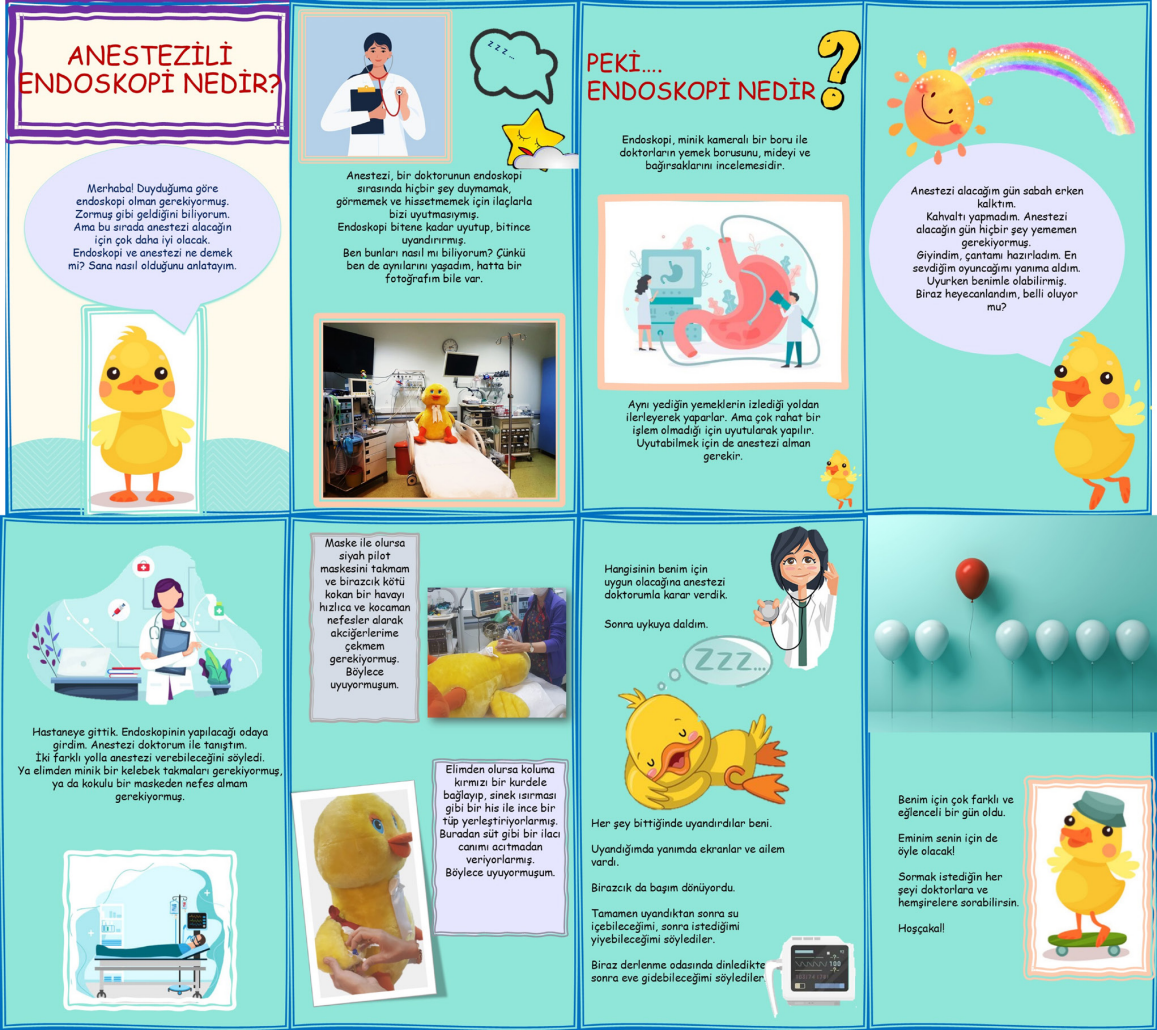
Snapshots of the illustrated educational brochure designed for children under 12. The brochure includes headings and explanations such as: “What is endoscopy with anesthesia?”, “So… what is endoscopy?”, “Anesthesia means that the doctor gives you medicines so you do not feel, hear, or see anything”, and “Endoscopy is the examination of the stomach and intestines with a small tube carrying a tiny camera.” It also describes the process in child-friendly language, for example: “On the day of anesthesia, I woke up early and did not eat breakfast”, “A small tube is placed into my hand through a needle”, “When I woke up, my doctors and family were beside me”, and “It was a very different and fun day for me”.

**Figure 3.**
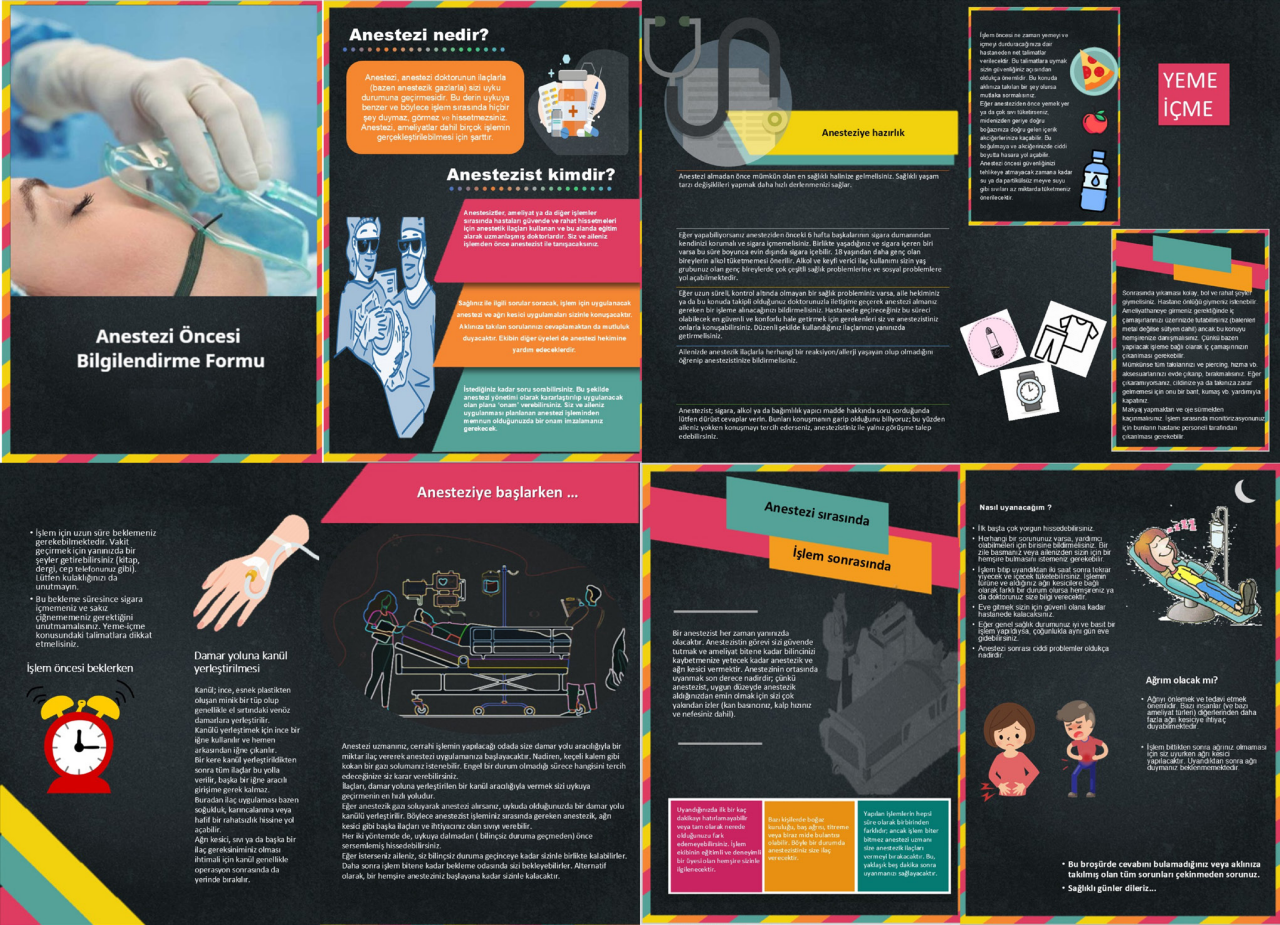
Snapshots of an educational brochure for children aged 12 and above, designed to provide age-appropriate guidance. The cover page is entitled “Pre-anesthesia information form”.

Two different educational brochures for children were developed, inspired by the Royal College of Anaesthetists’ Patient Information Resources.^17^ The first brochure, designed for children under 12 years of age, featured larger fonts, vibrant colors, and a duck character, which was also physically represented as a plush toy in the procedure and sedation areas to reinforce the provided information and offer emotional support. This brochure used age-appropriate visuals and simplified language to make the experience more relatable and reduce procedural anxiety (Figure 2). The second brochure, intended for children aged 12 years and older, contained more detailed educational text and fewer illustrations, tailored to meet the informational needs of older pediatric patients (Figure 3). Both brochures provided explanations about anesthesia and endoscopy, outlined preprocedural instructions, and described what to expect during the peri-sedation period. Additionally, a single educational brochure was prepared for caregivers, covering the same essential information.

### Anxiety Assessment and Sedation

The main outcome of the study was to compare the effect of educational brochure intervention on the anxiety scores of children and their caregivers undergoing outpatient gastroenterological endoscopic procedures with sedation. Consequently, evaluations of anesthesia-related knowledge and anxiety were deliberately performed on the day of the procedure, rather than prior to it, to better reflect the immediate preoperative psychological state.

On the day of the procedure, caregivers were asked to complete a survey assessing their demographic characteristics, knowledge of anesthesia and anxiety levels using the Amsterdam Preoperative Anxiety and Information Scale (APAIS).^18^ The APAIS responses were recorded on a 5-point Likert scale, where 1 indicated “strongly disagree” and 5 indicated “strongly agree.” The APAIS assessment was conducted approximately 30 minutes prior to the procedure, while the patients were waiting in the preprocedure holding area on the day of the procedure. The assessment was performed by a blinded anesthesiologist in the holding area. Patients or caregivers who did not read the educational brochure on the day of sedation were excluded from the study.

Prior to sedation, the children were placed in the procedure room and given time to familiarize themselves with the environment and anesthesia equipment. A plush duck toy was available at the bedside for those who wished to interact with it for comfort during sedation.

Pediatric anxiety was assessed preprocedure by a separate blinded investigator using the validated Turkish version of the Modified Yale Preoperative Anxiety Scale (m-YPAS). The m-YPAS has 5 domains: activity, vocalization, emotional expressivity, state of apparent arousal, and use of parents/caregivers.^19^ The total and corrected scores were calculated as: [(Activity + emotional expressivity + state of arousal + use of caregivers/4) + (vocalization/6)] × 100/5. A corrected m-YPAS score ≥30 was considered indicative of preprocedural anxiety.^19^

Sedation was administered under routine monitoring and induced either by sevoflurane inhalation or by intravenous administration of propofol, midazolam, or fentanyl, followed by propofol infusion with oxygen supplementation. After the procedure, the patients were transferred to the post-anesthesia care unit, where their caregivers were allowed to be with them. Patients were discharged when they were able to sit up unaided, swallow clear fluids without experiencing any sign of aspiration, and had an Aldrete score of ≥9, at least for a continuous hour.

To ensure blinding in the study, the anesthesiologists who distributed the educational brochures conducted the APAIS questionnaire in the interview room, performed the m-YPAS assessment, and administered sedation were all different individuals.

### Statistical Analysis

Statistical Package for Social Sciences (SPSS), version 22.0 (IBM SPSS Corp.; Armonk, NY, USA) was used for the statistical analyses of the study data. In the descriptive statistics section, categorical variables were evaluated as numbers and percentages and continuous variables were evaluated as mean ± standard deviation. The conformity of continuous variables to a normal distribution was evaluated using visual (histogram and probability graphs) and analytical methods (Kolmogorov–Smirnov/Shapiro–Wilk tests). For data that did not conform to a normal distribution, the Mann–Whitney *U* test was used for comparison between the 2 groups. Pearson’s chi-squared test was used to compare independent groups for categorical variables.

Binary logistic regression analysis was conducted to examine the effects of patient age, intervention (educational brochure), caregiver age, caregiver anxiety (APAIS score), and caregiver education level on preprocedural anxiety in children.

This study was approved by the Gazi University Faculty of Medicine Ethics Committee on December 13, 2021 (Approval no.: 184) and registered at ClinicalTrials.gov (Registration no.: NCT05221671) prior to participant enrollment. Informed consent was obtained from all caregivers involved in the study.

## Results

A total of 491 patients and their caregivers were assessed for eligibility, of whom 129 were excluded because they did not meet the inclusion criteria. Ultimately, 362 patients and caregivers were randomized into either the control group (n = 180) or intervention group (n = 182). However, 104 patients and/or caregivers did not read the educational brochure before the procedure and were excluded. Additionally, 6 patients/caregivers were lost to follow-up in the control group. The final number of participants was 78 in the intervention group and 174 in the control group, totaling 252 patients and their caregivers, respectively.

The mean patient age was 10.98 ± 4.45 years, while the mean caregiver age was 40.23 ± 6.92 years. Preprocedural pediatric anxiety, defined as m-YPAS ≥ 30, was observed in 51.6% of children. Of these, 79.2% were included in the control group. Patient age was categorized into preschool (3-5 years), school (6-11 years), and adolescent (12-17 years) groups. A Chi-square test examining the association between preprocedural anxiety and these age categories revealed a statistically significant difference in anxiety levels across age groups (*P* = .001).

Among caregivers, 74.2% were mothers, 21.4% were fathers, and 4.4% were other relatives. The most common educational attainment among caregivers was a university degree (34.5%), followed by middle school (27.8%), high school (7.0%), and a master’s degree (10.7%). Prior anesthesia experience was reported by 71.8% of children and 76.2% of caregivers.

Caregivers’ knowledge of anesthesia primarily stemmed from prior anesthesia experiences (55.6%), followed by information provided by their doctors (29.8%), relatives (6.3%), and news sources (4.4%). Regarding perceptions of anesthesia administration, 63.9% believed that it was delivered intravenously, 21.4% via inhalation, and 1.6% through local anesthesia, while 14.7% believed that all 3 methods were possible. Additionally, 7.9% reported having no prior knowledge of anesthesia delivery methods. Among caregivers, 36.1% reported fear of anesthesia. The most common concerns included the inability to wake up (45.6%), followed by nausea and vomiting (7.9%), sore throat (7.5%), death (6.7%), intraoperative awareness (4.4%), paralysis (4.0%), and pain (3.6%). The least feared complications were cough and respiratory distress, each cited by 0.4% of the caregivers.

The demographic characteristics of the control and intervention groups are presented in Table 1. Except for caregiver age, other demographic variables, including patient age, patient age category, caregiver identity, caregiver education level, and prior anesthesia experience of both patients and caregivers, showed no statistically significant differences. The presence of preprocedural anxiety was significantly higher in the control group that did not receive the educational brochure (*P* = .001). Preprocedural anxiety was identified in 130 children, of which 38.5% were school-aged and adolescents and 23.1% were preschoolers.

Binary logistic regression analysis was conducted to examine the effects of patient age category, intervention (educational brochure), caregiver age, caregiver APAIS score, and caregiver education level on preprocedural anxiety in children; the results are presented in Table 2. Preschool-aged children (3-5 years) were 8.1 times more likely to experience preprocedural anxiety than adolescents (OR = 8.10, 95% CI [2.92-22.42], *P* < .001). School-aged children (6-11 years) were 2.2 times more likely to experience preprocedural anxiety than adolescents (OR = 2.15, 95% CI [1.17-3.94], *P* = .01). Intervention with educational brochures was a significant factor in reducing preprocedural anxiety. Children in the control group were 3.69 times more likely to experience anxiety compared to those who received the educational brochure (OR = 3.691, 95% CI [1.96-6.92], *P* < .001). Caregiver factors, including caregiver age, educational status, and anxiety scores, were not significantly associated with pediatric preprocedural anxiety (*P* = .18, *P* = .30, and *P* = .96, respectively).

The anesthesia knowledge of the caregivers between the groups is presented in Table 3. There were no statistically significant differences between the study groups in the incidence of anesthesia-related fear, sources of anesthesia knowledge, perceived routes of anesthesia administration, or specific concerns regarding anesthesia (*P* > .05).

Caregivers’ APAIS scores for both groups are presented in Table 4. There were no statistically significant differences between the groups for any of the questions (*P* > .05). Parental educational status did not significantly impact parental anxiety scores (*P* = .6). The m-YPAS scores of the pediatric patients are shown in Table 5. There was a statistically significant difference between the study groups across all domains of the m-YPAS (*P* < .05).

Caregivers’ anxiety, measured by the APAIS, and pediatric patient anxiety, measured by the m-YPAS, are presented in Table 6. While the APAIS subdomain and total scores showed no statistically significant differences between the study groups, the m-YPAS scores demonstrated a significant difference (*P* < .001). Specifically, corrected m-YPAS scores were 14.1 points lower in the educational brochure group compared to the control group, indicating that children who received the educational brochure experienced significantly lower anxiety.

## Discussion

This randomized controlled trial investigated the efficacy of an age-appropriate educational brochure in mitigating preprocedural anxiety among pediatric patients and their caregivers undergoing endoscopy with sedation. A key finding was the significant reduction in anxiety observed in children who received the brochure, contrasting sharply with the lack of a similar effect among their caregivers. Concordantly, caregiver anxiety remained prevalent, with over a third expressing anesthesia-related concerns, most commonly the fear of their child “not waking up.” Furthermore, caregivers’ knowledge of anesthesia primarily stems from past personal experiences and information provided by medical doctors.

Children can experience especially high levels of anxiety before medical procedures.^4^In this study, nearly 60% of children who did not receive an informational brochure exhibited preprocedural anxiety. In contrast, providing educational brochures significantly reduces preprocedural anxiety in pediatric patients. The positive impact of the brochure on pediatric anxiety aligns with existing literature demonstrating the benefits of pre-anesthesia information, whether delivered through written, audiovisual, or verbal.^11^^,^^15^^,^^20^ One study even showed that a detailed verbal explanation of the periprocedural and sedation phases reduced anxiety, as confirmed by lower salivary cortisol in the intervention group.^21^ In this cohort of Turkish pediatric patients, the intervention successfully lowered anxiety, mirroring findings from studies using comic leaflets or detailed explanations.^15^^,^^16^ Notably, the design of the children’s brochure, particularly for the younger group, incorporated simplified language, engaging visuals featuring a character (a duck), and a corresponding plush toy available in the procedural setting. This age-tailored approach may have significantly contributed to the observed anxiety reduction in children, potentially offering a focal point for comfort and distraction that was absent in the caregiver intervention.

It is important to recognize that caregivers are not merely recipients of anxiety, but also reflectors of the uncertainty surrounding their child’s future and outcomes. Their distress often mirrors their fear of the unknown, emphasizing the need for interventions that address both their concerns and the underlying uncertainties they navigate. Reducing caregiver anxiety is crucial not only for mitigating children’s anxiety but also for ensuring better treatment compliance, as parental anxiety has been significantly associated with lower compliance rates.^22^Increased caregiver anxiety may lead to treatment delays and negatively affect pediatric outcomes. To address this issue, various non-pharmacological educational interventions have been developed.^23^^-^^25^^.^

Conversely, in this study the leaflet intervention did not significantly alter caregiver anxiety levels, and more than one-third of the caregivers reported fear of anesthesia. This finding resonates with the documented challenges in reducing parental anxiety through purely informational interventions.^26^ Although a range of educational formats—including short audiovisual modules, web-based games, and immersive virtual reality experiences—has been trialed, a universally accepted “gold-standard” intervention capable of reliably reducing caregiver anxiety has yet to be established.^23^^-^^25^The effectiveness of methods trialed to reduce parental anxiety varies, potentially due to differences in content, delivery method, timing, and assessment tools.^8^^-^^11^ Indeed, one trial observed higher caregiver anxiety after parents watched an educational video; the authors speculated that the clip was neither sufficiently simplified nor embedded within a counselling session that could contextualise the information and address emerging concerns.^25^ Taken together with the own finding that a written leaflet failed to lower parental distress, this result suggests that passive information transfer—whether textual or audiovisual—rarely addresses the complex emotional and cognitive components of caregiver anxiety. Multimodal, dialogue-rich interventions may therefore be required to achieve meaningful, durable reductions.

In this cohort, “not waking up after anesthesia” emerged—by a wide margin—as the chief parental fear. This pattern suggests limited anesthesia-specific health literacy: caregivers appear less aware of both common, benign events (e.g., postoperative nausea and vomiting) and rarer, yet more serious complications such as airway obstruction or perioperative mortality. The hierarchy of concerns clearly varies across sociocultural settings. One cross-sectional survey reported that >90% of mothers expressed anxiety in every anesthesia domain, spanning preoperative fasting, intravenous cannulation, and postoperative complaints including pain, nausea, vomiting, and even coma or death.^27^ By contrast, parents in this study focused almost exclusively on the single, catastrophic outcome of “not waking up,” underscoring a distinctive information gap. Taken together, these findings reinforce the need for culturally adapted, literacy-sensitive educational tools that address both the highly salient fear of permanent unconsciousness and the full spectrum of realistic anaesthetic risks.

The finding that most caregivers acquire their anesthesia knowledge from personal or acquaintances past operations highlights a persistent shortage of structured, standardised educational resources. Turkish studies demonstrated that formal counselling or written materials contribute little to patients’ perioperative understanding, a gap that is probably driven by limited clinic time for detailed explanations and sociocultural norms that privilege word-of-mouth learning.^28^^,^^29^Regardless of its origin, anecdotal information can entrench misconceptions, restrict true comprehension and, in turn, heighten preprocedural anxiety.

Addressing this deficit will require interactive, personalised strategies that move beyond a static brochure. Complementary options include brief audiovisual modules, scheduled question-and-answer sessions with anesthesia staff, and evidence-based behavioral or mindfulness techniques—each adaptable to individual caregiver needs. ^8^^,^^30^^-^^33^To maximise real-world impact, these tools could be delivered in digital form (mobile apps, interactive websites), embedded systematically within pre-anaesthetic clinic visits, and, where appropriate, supplemented by child-life specialists or psychologists who can provide targeted support to highly anxious families. Such multimodal, culturally attuned approaches are more likely to correct misinformation, enhance informed decision-making and ultimately reduce anxiety for both children and their caregivers.

Pediatric anxiety is influenced not only by child-specific factors and parenteral anxiety but also by various caregiver-related characteristics, such as caregiver educational level.^11^^-^^16^^,^^20^^,^^22^^-^^26^^,^^34^^-^^36^ However, the study did not observe any significant association between preprocedural pediatric anxiety and caregiver educational level. While this finding aligns with some studies in the literature, the lack of correlation between caregiver education level and anxiety is noteworthy. This is corroborated by Çağiran et al, who found that maternal sociodemographic characteristics, including education, did not significantly influence children’s anxiety levels during surgical procedures.^37^ Similarly, studies emphasizing the need for effective communication and education underscore that increasing parental knowledge may not inherently lead to reduced anxiety, pointing to the potential influence of factors such as situational contexts, and the child’s specific experiences.^38^^,^^39^ This may suggest that health literacy levels or sociocultural factors and differences in parenting strategies could influence anxiety levels independently of formal education.^37^^,^^39^ How parents process information and interact with healthcare systems might be more determinant of their anxiety levels than their educational attainment.

### Limitations

This study has several limitations. First, anxiety was assessed only once—immediately before the procedure—without an earlier baseline measurement. Although the trial was deliberately designed to compare the brochure and no-brochure groups rather than pre-to-post changes within individuals, the absence of a true baseline prevents quantification of the brochure’s absolute effect. Second, recording the vital parameters of both the children and caregivers, as well as anesthetic drug dosages during the procedure, could have provided additional physiological insights into anxiety-related responses. Third, the inclusion of children undergoing endoscopy for patients with nonurgent gastrointestinal bleeding may have introduced clinical heterogeneity, because even though it is non urgent bleeding, stress of being chronic cases differ from purely elective indications. Finally, a long-term post-procedural follow-up of families would have allowed for a more comprehensive evaluation of the intervention’s lasting effects. Gathering long-term follow-up data in future studies is important to understand whether the reduction in post-procedural anxiety persists over time or if additional support is needed.

Providing children with age-appropriate educational brochures before endoscopic sedation significantly reduced perioperative anxiety, although it did not have a notable effect on caregiver anxiety. This low-cost and easily implemented intervention may improve procedural experience in pediatric patients and potentially lead to better overall clinical outcomes.

## Figures and Tables

**Table 1.** Demographic Characteristics of Study Groups

		Control Group (n = 174)	Educational Brochure Group (n = 78)	*P*
Patient age (mean ± SD)*		10.55 ± 4.648	11.17 ± 4.360	.30
Caregiver age (mean ± SD)*		38.74 ± 6.706	40.90 ± 6.926	**.022**
Patient age group in years, n (%)**	Preschool (3-5)	22 (12.6)	15 (19.2)	.38
	School (6-11)	62 (35.6)	25 (32.1)	
	Adolescent (12-17)	90 (51.7)	38 (48.7)	
Caregiver identity, n (%)**	Mother	131 (75.3)	56 (71.8)	.61
	Father	35 (20.1)	19 (24.4)	
	Other relatives	8 (4.6)	3 (3.9)	
Caregiver education level, n (%)**	Middle school	51 (29.3)	19 (24.4)	.32
	High school	48 (27.6)	20 (25.6)	
	University	54 (31.0)	33 (42.3)	
	Master degree	21 (12.1)	6 (7.7)	
Caregiver prior anesthesia experience, n (%)**	Yes	131 (75.3)	61 (78.2)	.615
	No	43 (24.7)	17 (21.8)	
Patient prior anesthesia experience, n (%)**	Yes	126 (72.4)	55 (70.5)	.76
	No	48 (27.6)	23 (29.5)	
Preprocedural pediatric anxiety, n (%)**	Yes	103 (59.2)	27 (34.6)	**.001**
	No	71 (40.8)	51 (65.4)	

SD, standard deviation.

*Significance level obtained with Mann–Whitney *U*.

**Significance level obtained with Pearson chi-square.

**Table 2.** Effect of Predictors on Pediatric Preprocedural Anxiety

	B	S.E.	Wald	*df*	Odds Ratio (95% CI)	*P* ^&^
Patient age group						
	Adolescent
	N/A	N/A	17.913	2	N/A	<.001
	Preschool
2.092	0.519	16.226	1	8.102 (2.927-22.422)	<.001
School
0.768	0.308	6.223	1	2.155 (1.179-3.940)	.01
Educational brochure	1.306	0.321	16.517	1	3.691 (1.966-6.927)	<.001
Caregiver age	−0.030	0.022	1.770	1	0.971 (0.929-1.014)	.18
Caregiver APAIS, total score	−0.001	0.025	0.002	1	0.999 (0.952-1.048)	.96
Caregiver educational attainment						
	Masters
	N/A	N/A	1.487	3	N/A	.68
	Middle school
	0.185	0.497	0.139	1	1.204 (0.455-3.187)	.71
High school
0.330	0.505	0.427	1	1.391 (0.517-3.745)	.51
University
−0.092	0.490	0.036	1	0.912 (0.349-0.238)	.85

APAIS, anxiety and preoperative information scale.

^&^Significance levels derived from binary logistic regression analysis.

**Table 3.** Anesthesia Knowledge of Caregivers Between Groups

		Control Group (n = 174) (%)	Educational Brochure Group (n = 78) (%)	P**
Fear of anesthesia	Yes	65 (37.4)	26 (33.3)	.54
	No	109 (62.6)	52 (66.7)	
Source of anesthesia knowledge	Prior anesthesia	101 (58)	39 (50)	.24
	Doctors	46 (26.4)	29 (37.2)	.09
	Relatives	11 (6.3)	5 (6.4)	.98
	News	10 (5.7)	1 (1.3)	.11
	Other sources	19 (10.9)	14 (17.9)	.13
Anesthesia delivery routes	Intravenous	111 (63.8)	50 (64.1)	.96
	Inhalation	32 (18.4)	22 (28.2)	.80
	Local	4 (2.3)	0 (0)	.18
	All of the above	25(14.4)	12 (15.4)	.83
	No knowledge	16 (9.2)	4 (5.1)	.27
Concerns regarding anesthesia	Inability to wake up	79 (45.4)	36 (46.2)	.91
	Nausea/vomiting	14 (8)	6 (7.7)	.93
	Sore throat	11 (6.3)	8 (10.3)	.27
	Death	13 (7.5)	4 (5.1)	.49
	Intraoperative awareness	7 (4)	4 (5.1)	.69
	Paralysis	8 (4.6)	2 (2.6)	.45
	Pain	5 (2.9)	4 (5.1)	.37
	Cough	1 (0.6)	0 (0)	.50
	Respiratory distress	1 (0.6)	0 (0)	.50
	All of the above	1 (0.6)	0 (0)	.50
	No concern	58 (33.3)	28 (35.9)	.69

**Significance level obtained with Pearson chi-square.

**Table 4.** Caregiver APAIS Between Study Groups

Questions Related to Anesthesia	Control Group Median (IQR)	Educational Brochure Group Median (IQR)	P*
I am worried about anesthesia	3 (1-4)	3 (2-4)	.53
The anesthesia is constantly on my mind	1 (1-3)	1 (1-3)	.64
I would like to know as much as possible about the anesthesia	4 (3-5)	4 (2-5)	1.0
**Questions Related to Procedure**	**Control Group ** **Median (IQR)**	**Educational Brochure Group ** **Median (IQR)**	P*
I am worried about the procedure	3 (1-5)	3 (1.75-4)	.29
The procedure is constantly on my mind	3 (1-4)	2 (1-4)	.81
I would like to know as much as possible about the procedure	4.5 (3-5)	4.5 (3-5)	.87

APAIS, anxiety and preoperative information scale; IQR, interquartile range.

*Significance level obtained with Mann–Whitney *U*.

**Table 5.** m-YPAS Scores Between Study Groups

m-YPAS Domains	Control Group Median (IQR)	Educational Brochure Group Median (IQR)	P*
Activity	1 (1-2)	1 (1-1)	.001
Vocalization	1 (1-3)	1 (1-1)	.005
Emotional expressivity	2 (1-3)	1 (1-2)	.001
State of apparent arousal	1 (1-2)	1 (1-2)	.017
Use of parents/caregivers	1 (1-2)	1 (1-1)	.012

IQR, interquartile range; m-YPAS, modified Yale preoperative anxiety scale.

*Significance level obtained with Mann–Whitney *U*.

**Table 6.** Anxiety Measurements of Caregivers and Pediatric Patients Between Groups

Anxiety Measurements	Control Group Median (IQR)	Educational Brochure Group Median (IQR)	P*
Caregiver APAIS, anesthesia-related questions	9 (6-11)	9 (6-10)	.46
Caregiver APAIS, procedure-related questions	10 (7-12)	9 (7-11.25)	.55
Caregiver APAIS, total	18 (14-23)	18 (14-21.25)	.41
Pediatric m-YPAS, total	8 (5-12)	5 (5-9)	**.001**
Pediatric, m-YPAS, corrected total	37.5 (23.3-55.0)	23.3 (23.3-40.4)	**<.001**

APAIS, anxiety and preoperative information scale; IQR, interquartile range; m-YPAS, modified Yale preoperative anxiety scale.

*Significance level obtained with Mann–Whitney *U*.

## Data Availability

Authors agree to make data and materials supporting the results or analyses presented in their paper available upon reasonable request.
